# Supplementary material for: The maternal postnatal six-week check in women with epilepsy: Does the prevalence or subsequent postpartum health differ from the general postnatal population?
Source: PLoS One. 2025 May 30;20(5):e0323135. doi: 10.1371/journal.pone.0323135 (PMC12124846; doi:10.1371/journal.pone.0323135)
Supplement: S1 Table — (DOCX) [file pone.0323135.s001.docx]

**S1 Table. Derivation of data items of interest**

|  | **Derivation** |
| --- | --- |
| **Live birth or stillbirth** | Since 1992, a stillbirth has been defined in the UK as a baby born dead after 24 weeks of pregnancy while a miscarriage includes all pregnancy losses from the time of conception until 24 weeks of pregnancy. In this study, a live birth or stillbirth was identified on the basis of having a CPRD pregnancy register outcome of 1, 2, 3, 11 or 12 OR having any ICD-10 or OPCS delivery related codes in HES APC or valid entry in a delivery related field in HES maternity (see the following files in [GitHub](https://github.com/Fitzpatrickk/CPRD-Aurum-HES-codes_maternal-six-week-check_epilepsy_pregnancy/tree/main): ‘ICD-10_delivery codes’, ‘OPCS_delivery codes’ and `HES_Maternity_values_delivery’). See S1 Fig for details about how overlapping and implausibly close pregnancy records were dealt with. More details about the CPRD pregnancy register can be found in <https://www.cprd.com/sites/default/files/2022-05/CPRD%20Aurum%20PregnancyRegister_Documentation_v1.pdf> |
| **Epilepsy** | Epilepsy diagnosis codes in primary care records at any time up to date gave birth. Codes used found in ‘Epilepsy’ file in [GitHub](https://github.com/Fitzpatrickk/CPRD-Aurum-HES-codes_maternal-six-week-check_epilepsy_pregnancy/tree/main). |
| **Maternal SWC** | Codes in primary care records specifically describing maternal SWCs or had codes indicating a possible maternal SWC such as ‘postnatal examination observations’ between 4-12 weeks postpartum. Codes used can be found in ‘Maternal SWC’ file in [GitHub](https://github.com/Fitzpatrickk/CPRD-Aurum-HES-codes_maternal-six-week-check_epilepsy_pregnancy/tree/main). |
| **A&E visit for epilepsy** | HES A&E diagnosis code for epilepsy (241) |
| **Unplanned hospital admission for epilepsy** | ICD-10 code in HES APC for epilepsy (G40, G41) and method of admission unplanned (admimeth 21-25, 28, 2A, 2B, 2C or 2D) |
| **Mortality** | Record in ONS death registration data or according to primary care records (cprd_ddate) |
| **Pregnancy prevention plan** | Pregnancy prevention plan codes in primary care records. Codes used found in ‘Pregnancy prevention plan” file in [GitHub](https://github.com/Fitzpatrickk/CPRD-Aurum-HES-codes_maternal-six-week-check_epilepsy_pregnancy/tree/main). |
| **Sodium valproate** | Prescription for sodium valproate in primary care records. Codes used found in ‘Valproate prescription’ file in [GitHub](https://github.com/Fitzpatrickk/CPRD-Aurum-HES-codes_maternal-six-week-check_epilepsy_pregnancy/tree/main). |
| **Prescribed prophylactic contraception** | Prescription for prophylactic contraception in primary care records. Codes used found in ‘Prophylactic contraception’ file in [GitHub](https://github.com/Fitzpatrickk/CPRD-Aurum-HES-codes_maternal-six-week-check_epilepsy_pregnancy/tree/main). |
| **Prescribed emergency contraception** | Prescription for emergency contraception in primary care records. Codes used found in ‘Emergency contraception’ file in [GitHub](https://github.com/Fitzpatrickk/CPRD-Aurum-HES-codes_maternal-six-week-check_epilepsy_pregnancy/tree/main). |
| **Cannot become pregnant** | Codes in primary care records indicating the woman could not become pregnant. Codes used can be found in “Cannot become pregnant’ file in [GitHub](https://github.com/Fitzpatrickk/CPRD-Aurum-HES-codes_maternal-six-week-check_epilepsy_pregnancy/tree/main). |
| **Depression &/or anxiety** | Depression and/or anxiety diagnosis codes in primary care records OR depression and/or anxiety symptom codes with a prescription for antidepressants and/or antianxiety medication within 4 weeks of symptoms in primary care records. Codes used can be found in ‘Depression_anxiety diagnosis’, ‘Depression_anxiety symptoms” and ‘Medication depression_anxiety’ files in [GitHub](https://github.com/Fitzpatrickk/CPRD-Aurum-HES-codes_maternal-six-week-check_epilepsy_pregnancy/tree/main). |
| **Urinary &/or faecal incontinence** | Codes for urinary and/or faecal incontinence in primary care records. Codes used can be found in ‘Urinary_faecal incontience’ file in [GitHub](https://github.com/Fitzpatrickk/CPRD-Aurum-HES-codes_maternal-six-week-check_epilepsy_pregnancy/tree/main). |
| **Dyspareunia, perineal &/or pelvic pain** | Codes for dyspareunia, perineal &/or pelvic pain in primary care records. Codes used can be found in ‘Dyspareunia_perineal_pelvicpain’ file in [GitHub](https://github.com/Fitzpatrickk/CPRD-Aurum-HES-codes_maternal-six-week-check_epilepsy_pregnancy/tree/main). |
| **Age at delivery** | Derived from women’s year and month of birth recorded in primary care records and date gave birth according to CPRD pregnancy register or HES APC. |
| **Ethnic group** | Based on ethnicity codes recorded in primary care records (see ‘Ethnicity’ file in [GitHub](https://github.com/Fitzpatrickk/CPRD-Aurum-HES-codes_maternal-six-week-check_epilepsy_pregnancy/tree/main)) OR according to ethnos field in HES APC if no ethnicity codes in primary care records. Where a woman had multiple ethnicity records with different ethnic categories recorded, a previously published algorithm(1) was used to identify the single most plausible ethnic category, based on prioritising records by frequency, then recentness, then the ethnicity most commonly occurring in the population. |
| **Geographic region** | Geographic region of the GP practice |
| **IMD** | IMD corresponding to the woman’s postcode of residence |
| **Parity** | Based on numpreg field in HES APC OR number of previous births observed in HES and CPRD pregnancy register if numpreg missing or numpreg less than the number of previous births observed. |
| **Multifetal pregnancy** | ICD-10 delivery related codes in HES APC indicating a multiple birth (see ‘ICD-10_delivery codes’ file in [GitHub](https://github.com/Fitzpatrickk/CPRD-Aurum-HES-codes_maternal-six-week-check_epilepsy_pregnancy/tree/main)) OR woman had episodes in HES Maternity with the same start and end dates but different values of birthweight that were not missing OR evidence of multiple pregnancy according to the CPRD pregnancy register. |
| **Gestational hypertension or pre-eclampsia** | Codes for gestational hypertension or pre-eclampsia from 20 week gestation up to 6 week postpartum in primary care records (see ‘PIH_pre-eclampsia’ file in [GitHub](https://github.com/Fitzpatrickk/CPRD-Aurum-HES-codes_maternal-six-week-check_epilepsy_pregnancy/tree/main)) or HES APC (ICD-10 codes O11, O13-O15) OR codes for possible gestational hypertension or pre-eclampsia from 20 week gestation up to 6 week postpartum in primary care records (see ‘Possible_PIH_pre-eclampsia’ file in [GitHub](https://github.com/Fitzpatrickk/CPRD-Aurum-HES-codes_maternal-six-week-check_epilepsy_pregnancy/tree/main)) or HES APC (ICD-10 code 016) if no codes for pre-existing hypertension before 20 weeks gestation up to 1 year prior to pregnancy in primary care records (see ‘Pre-existing hypertension’ file in [GitHub](https://github.com/Fitzpatrickk/CPRD-Aurum-HES-codes_maternal-six-week-check_epilepsy_pregnancy/tree/main)) or HES APC (I10-I15, O10). |
| **Mode of birth** | Based on OPCS mode of birth codes in HES (see ‘OPCS_delivery codes’ file in [GitHub](https://github.com/Fitzpatrickk/CPRD-Aurum-HES-codes_maternal-six-week-check_epilepsy_pregnancy/tree/main) OR delmeth field in HES Maternity if no OPCS mode of birth codes. If >1 mode of birth code, prioritised most invasive mode of birth (emergency caesarean followed by elective caesarean, assisted vaginal birth, unassisted vaginal birth, other) |
| **Preterm birth** | Gestational age at birth <37 weeks according to gestat field in HES Maternity OR according to gestdays field in CPRD pregnancy register OR derived from pregnancy start and ends dates (see S1 Fig for more details). Gestational age values of <24 weeks or >43 weeks regarded as implausible and recoded as missing. |
| **Number of GP contacts in the year before pregnancy** | Based on primary care consultation records, counting consultation records in the year before pregnancy involving interaction between patient and practice (not necessarily face to face) determined using codes found in ‘consmedcodeid_GPcontact’ and ‘Conssourceid_GPcontact’ files in [GitHub](https://github.com/Fitzpatrickk/CPRD-Aurum-HES-codes_maternal-six-week-check_epilepsy_pregnancy/tree/main), and allowing a maximum of 1 consultation per day. |

Abbreviations: A&E, Accident and Emergency; APC, Admitted Patient Care; CPRD, Clinical Practice Research Datalink; GP, General practitioner; HES, Hospital Episode Statistics; ICD-10, International Classification of Diseases 10^th^ revision; IMD, Index of Multiple Deprivation; OPCS, Office of Population Censuses and Surveys Classification of Surgical Operations and Procedures; SWC, postnatal six-week check

1. Zhang CX, Bankhead C, Quigley MA, Kwok CH, Carson C. Ethnic inequities in routine childhood vaccinations in England 2006-2021: an observational cohort study using electronic health records. EClinicalMedicine. 2023;65:102281.
