# Supplementary material for: The maternal postnatal six-week check in women with epilepsy: Does the prevalence or subsequent postpartum health differ from the general postnatal population?
Source: PLoS One. 2025 May 30;20(5):e0323135. doi: 10.1371/journal.pone.0323135 (PMC12124846; doi:10.1371/journal.pone.0323135)
Supplement: S1 Fig — (PDF) [file pone.0323135.s002.pdf]

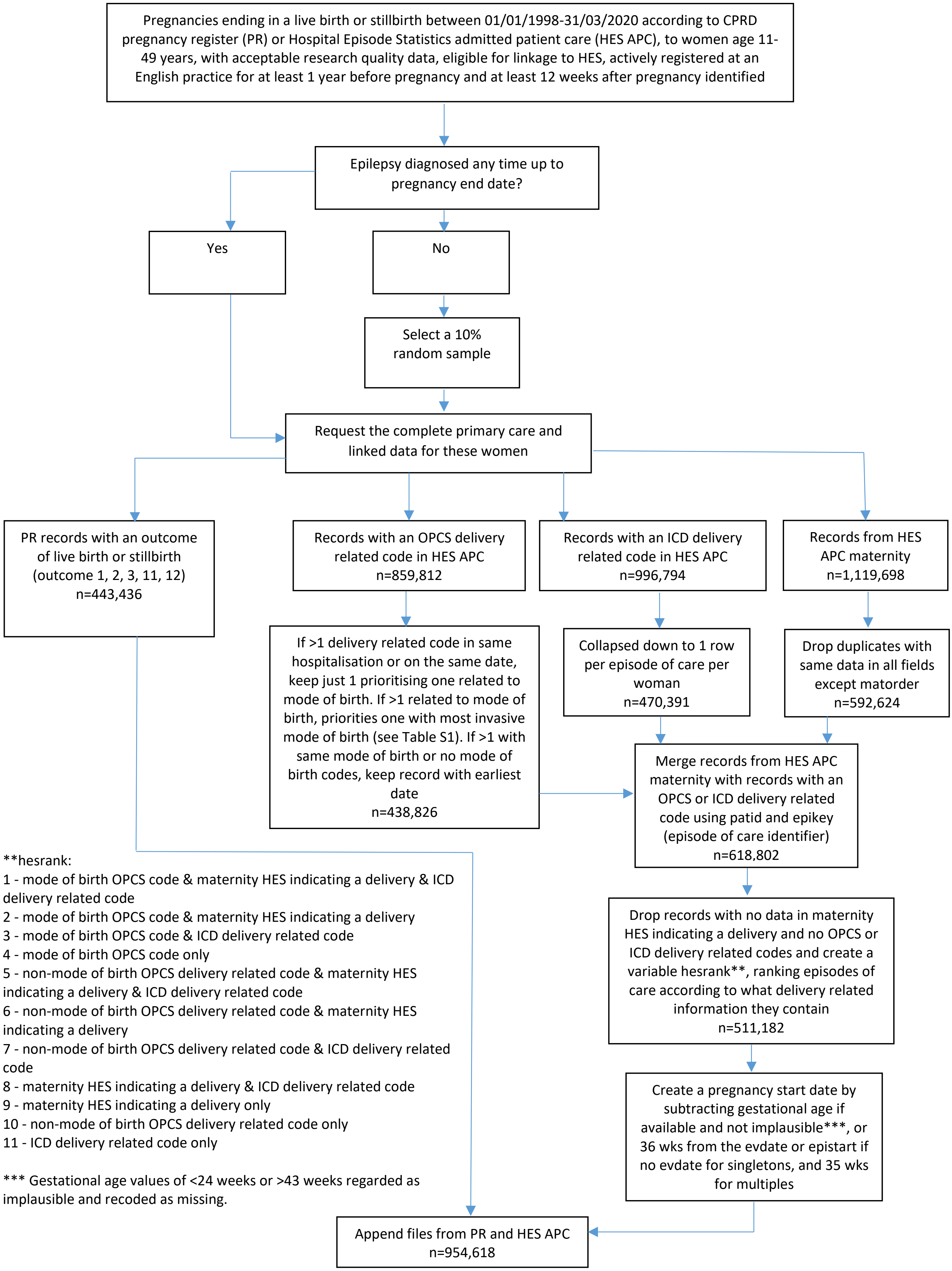

S1 Fig. Flow diagram of identification of study population – part1

(continued on next page)

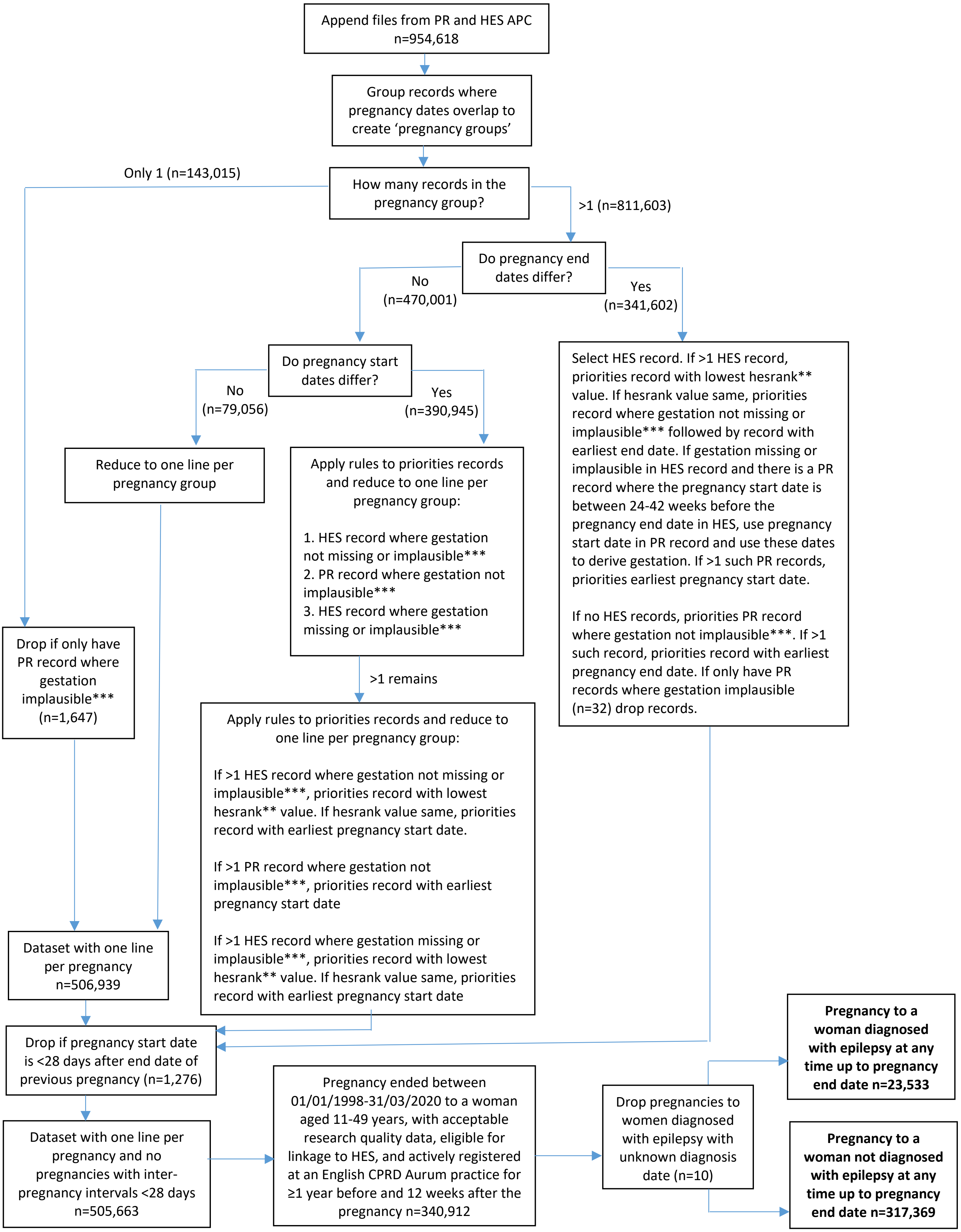

S1 Fig. Flow diagram of identification of study population – part 2
