# Supplementary material for: The maternal postnatal six-week check in women with epilepsy: Does the prevalence or subsequent postpartum health differ from the general postnatal population?
Source: PLoS One. 2025 May 30;20(5):e0323135. doi: 10.1371/journal.pone.0323135 (PMC12124846; doi:10.1371/journal.pone.0323135)
Supplement: S2 Table — (DOCX) [file pone.0323135.s003.docx]

**S2 Table. Characteristics of complete case and whole study population**

|  | **Complete case**  **n (%) study population**  **N=293,272** | **Whole**  **n (%)* study population**  **N=340,902** |
| --- | --- | --- |
| ***Maternal socio-demographic characteristics*** | | |
| **Age at delivery in years** |  |  |
| <20 | 12,692 (4.3) | 15,235 (4.5) |
| 20-24 | 44,660 (15.2) | 51,137 (15.0) |
| 25-29 | 78,050 (26.6) | 89,529 (26.3) |
| 30-34 | 92,636 (31.6) | 107,242 (31.5) |
| 35-39 | 53,363 (18.2) | 62,536 (18.3) |
| ≥40 | 11,871 (4.0) | 15,223 (4.5) |
| **Median (IQR) age at delivery in years** | 30.6 (26.2,34.5) | 30.7 (26.3,34.6) |
| **Ethnic group** |  |  |
| White | 243,048 (82.9) | 279,830 (83.1) |
| Asian or Asian British | 28,890 (9.9) | 32,411 (9.6) |
| Black, African, Caribbean or Black British | 12,874 (4.4) | 14,983 (4.4) |
| Mixed or multiple ethnic groups | 3,898 (1.3) | 4,471 (1.3) |
| Other ethnic group | 4,562 (1.6) | 5,149 (1.5) |
| *Missing (%)* | *-* | *1.2* |
| **Geographic region** |  |  |
| North East, Yorkshire and the Humber | 20,453 (7.0) | 22,974 (6.7) |
| North West | 58,050 (19.8) | 66,787 (19.6) |
| Midlands | 58,170 (19.8) | 66,057 (19.4) |
| East of England | 12,682 (4.3) | 14,700 (4.3) |
| London | 50,966 (17.4) | 60,984 (17.9) |
| South East | 57,398 (19.6) | 67,500 (19.8) |
| South West | 35,553 (12.1) | 41,900 (12.3) |
| **IMD** |  |  |
| 1 (least deprived) | 53,156 (18.1) | 62,425 (18.4) |
| 2 | 52,843 (18.0) | 61,624 (18.1) |
| 3 | 52,171 (17.8) | 60,812 (17.9) |
| 4 | 60,914 (20.8) | 70,120 (20.6) |
| 5 (most deprived) | 74,188 (25.3) | 84,756 (24.9) |
| *Missing (%)* | *-* | *0.3* |
| ***Pregnancy/birth characteristics*** | | |
| **Parity**^d^ |  |  |
| 0 | 87,806 (29.9) | 104,234 (30.6) |
| ≥1 | 205,466 (70.1) | 236,668 (69.4) |
| **Multifetal pregnancy** | 4,393 (1.5) | 4,927 (1.4) |
| **Gestational hypertension or pre-eclampsia** | 21,643 (7.4) | 23,523 (6.9) |
| **Mode of birth** |  |  |
| Emergency caesarean section | 37,695 (12.9) | 40,110 (12.8) |
| Elective caesarean section | 33,058 (11.3) | 34,929 (11.2) |
| Assisted vaginal birth | 31,361 (10.7) | 33,527 (10.7) |
| Unassisted vaginal birth | 190,730 (65.0) | 203,749 (65.1) |
| Other | 428 (0.1) | 663 (0.2) |
| *Missing (%)* | *-* | *8.2* |
| **Preterm birth (<37 weeks of gestation)**^h^ | 22,545 (7.7) | 24,192 (7.5) |
| *Missing (%)* | *-* | *5.1* |
| ***Prior health care utilisation/medical history*** | | |
| **Number of GP contacts in the year before pregnancy** |  |  |
| 0 | 42,695 (14.6) | 54,516 (16.0) |
| 1-3 | 86,357 (29.4) | 99,471 (29.2) |
| 4-9 | 108,446 (37.0) | 123,136 (36.1) |
| ≥10 | 55,774 (19.0) | 63,779 (18.7) |
| **Median (IQR) number of GP contacts in the year before pregnancy** | 4.0 (2.0,8.0) | 4.0 (1.0,8.0) |
| **Prescribed prophylactic contraception at any point between the year before pregnancy and prior to the SWC or index date** | 110,456 (37.7) | 127,329 (37.4) |
| **Prescribed emergency contraception at any point between the year before pregnancy and prior to the SWC or index date** | 9,334 (3.2) | 10,616 (3.1) |
| **Depression &/or anxiety diagnosis at any point between the year before pregnancy and prior to the SWC or index date** | 26,454 (9.0) | 30,633 (9.0) |
| **Urinary &/or faecal incontinence at any point between the year before pregnancy and prior to the SWC or index date** | 2,287 (0.8) | 2,705 (0.8) |
| **Dyspareunia, perineal &/or pelvic pain at any point between the year before pregnancy and prior to the SWC or index date** | 7,232 (2.5) | 8,155 (2.4) |

**^a^** Percentage of those with complete data

Abbreviations: GP, General practitioner; IMD, Index of Multiple Deprivation; IQR, interquartile range; SWC, postnatal six-week check
