# Supplementary material for: The maternal postnatal six-week check in women with epilepsy: Does the prevalence or subsequent postpartum health differ from the general postnatal population?
Source: PLoS One. 2025 May 30;20(5):e0323135. doi: 10.1371/journal.pone.0323135 (PMC12124846; doi:10.1371/journal.pone.0323135)
Supplement: S3 Table — (DOCX) [file pone.0323135.s004.docx]

**S3 Table. General postpartum health outcomes in those who did not compared to did have a maternal SWC by epilepsy status**

| **Outcome** | **Epilepsy status** | **Had a maternal SWC**  **number of events/ person-years (rate per 100 person-years)** | **Did not have a maternal SWC number of events/ person-years (rate per 100 person-years)** | **aHR* (95% CI)** | **P-value for interaction between maternal SWC and whether or not a women had epilepsy** |
| --- | --- | --- | --- | --- | --- |
| Prescribed prophylactic contraception | Epilepsy | 7,237/5,893 (122.81) | 3,838/5,854  (65.56) | **0.60 (0.58-0.63) p<0.001** | 0.202 |
|  | No epilepsy | 96,294/78,833 (122.15) | 51,550/82,569 (62.43) | **0.59 (0.58-0.60) p<0.001** |  |
| Prescribed emergency contraception | Epilepsy | 407/11,006  (3.70) | 283/8,152  (3.47) | 1.0 (0.85-1.17) p=0.973 | 0.534 |
|  | No epilepsy | 5,162/148,350 (3.48) | 3,560/113,380 (3.14) | **0.95 (0.90-0.99) p=0.020** |  |
| Depression &/or anxiety | Epilepsy | 1,875/10,296 (18.21) | 1,297/7,688  (16.87) | 1.01 (0.93-1.09) p=0.815 | **<0.001** |
|  | No epilepsy | 18,665/141,740 (13.17) | 11,806/109,680 (10.76) | **0.86 (0.84-0.89) p<0.001** |  |
| Urinary &/or faecal incontinence | Epilepsy | 164/11,232  (1.46) | 70/8,346  (0.84) | **0.68 (0.49-0.93) p=0.016** | 0.943 |
|  | No epilepsy | 1,780/150,870 (1.18) | 798/115,480  (0.69) | **0.67 (0.61-0.73) p<0.001** |  |
| Dyspareunia, perineal &/or pelvic pain | Epilepsy | 242/11,205  (2.16) | 113/8,325  (1.36) | **0.75 (0.59-0.95) p=0.018** | 0.565 |
|  | No epilepsy | 2,654/150,620 (1.76) | 1,205/115,310 (1.04) | **0.69 (0.64-0.75) p<0.001** |  |

*adjusted for year of birth, maternal socio-demographic characteristics (age at delivery, ethnic group, geographic region & IMD), pregnancy/birth characteristics (parity, multifetal pregnancy, gestational hypertension or pre-eclampsia, mode of birth & preterm birth), and prior health care utilisation/medical history (number of GP contacts in the year before pregnancy and whether outcome in question was recorded at any point between the year before pregnancy and prior to the SWC or index date)

Abbreviations: CI, confidence interval; GP, General practitioner; HR, Hazard ratio; IMD, Index of Multiple Deprivation; SWC, postnatal six-week check
