# Supplementary material for: The maternal postnatal six-week check in women with epilepsy: Does the prevalence or subsequent postpartum health differ from the general postnatal population?
Source: PLoS One. 2025 May 30;20(5):e0323135. doi: 10.1371/journal.pone.0323135 (PMC12124846; doi:10.1371/journal.pone.0323135)
Supplement: Attachment — Submitted filename: Response to Reviewers.docx [file pone.0323135.s006.docx]

**Response to Reviewers**

**Journal Requirements:**

1. When submitting your revision, we need you to address these additional requirements.

Please ensure that your manuscript meets PLOS ONE's style requirements, including those for file naming. The PLOS ONE style templates can be found at

<https://journals.plos.org/plosone/s/file?id=wjVg/PLOSOne_formatting_sample_main_body.pdf> and

<https://journals.plos.org/plosone/s/file?id=ba62/PLOSOne_formatting_sample_title_authors_affiliations.pdf>

**We have now ensured that our manuscript meets the PLOS ONE’s style requirements.**

2. Please note that PLOS ONE has specific guidelines on code sharing for submissions in which author-generated code underpins the findings in the manuscript. In these cases, we expect all author-generated code to be made available without restrictions upon publication of the work. Please review our guidelines at <https://journals.plos.org/plosone/s/materials-and-software-sharing#loc-sharing-code> and ensure that your code is shared in a way that follows best practice and facilitates reproducibility and reuse.

**A complete description of the data sources, codes, and database fields used is provided in the supplementary information (S1 Table), which includes a link to the GitHub repository containing all the code lists used to derive data items.**

3. Thank you for stating the following financial disclosure:

 This research is funded by the National Institute for Health Research (NIHR) Policy Research Programme, conducted through the NIHR Policy Research Unit in Maternal and Neonatal Health and Care, PR-PRU-1217-21202. SK is part funded by the NIHR Applied Research Collaboration in the West Midlands. The views expressed are those of the authors and not necessarily those of the NIHR or the Department of Health and Social Care.

Please state what role the funders took in the study.  If the funders had no role, please state: "The funders had no role in study design, data collection and analysis, decision to publish, or preparation of the manuscript."

If this statement is not correct you must amend it as needed.

Please include this amended Role of Funder statement in your cover letter; we will change the online submission form on your behalf.

**We have also included the financial disclosure statement with the amended Role of Funder statement at the bottom of our cover letter as requested.**

4. We note that you have indicated that there are restrictions to data sharing for this study. For studies involving human research participant data or other sensitive data, we encourage authors to share de-identified or anonymized data. However, when data cannot be publicly shared for ethical reasons, we allow authors to make their data sets available upon request. For information on unacceptable data access restrictions, please see <http://journals.plos.org/plosone/s/data-availability#loc-unacceptable-data-access-restrictions>.

Before we proceed with your manuscript, please address the following prompts:

a) If there are ethical or legal restrictions on sharing a de-identified data set, please explain them in detail (e.g., data contain potentially identifying or sensitive patient information, data are owned by a third-party organization, etc.) and who has imposed them (e.g., a Research Ethics Committee or Institutional Review Board, etc.). Please also provide contact information for a data access committee, ethics committee, or other institutional body to which data requests may be sent.

b) If there are no restrictions, please upload the minimal anonymized data set necessary to replicate your study findings to a stable, public repository and provide us with the relevant URLs, DOIs, or accession numbers. Please see <http://www.bmj.com/content/340/bmj.c181.long> for guidelines on how to de-identify and prepare clinical data for publication. For a list of recommended repositories, please see <https://journals.plos.org/plosone/s/recommended-repositories>. You also have the option of uploading the data as Supporting Information files, but we would recommend depositing data directly to a data repository if possible.

Please update your Data Availability statement in the submission form accordingly.

**We have updated our data availability statement to the following: This study (protocol number 22_002473) was approved through the CPRD Research Data Governance process. All data used in this study were provided by the CPRD. CPRD data governance and the license to use CPRD does not allow the data to be shared. Researchers must seek approval via CPRD’s Research Data Governance process to access the data for the purposes of their own research (https://www.cprd.com/data-access). This study is based in part on data from the CPRD obtained under licence from the UK Medicines and Healthcare products Regulatory Agency. The data is provided by patients and collected by the NHS as part of their care and support. Linked data were also provided by the ONS. ONS and HES data © (2021) were re-used with the permission of The Health & Social Care Information Centre. All rights reserved. The interpretation and conclusions contained in this study are those of the authors alone.**

5. Please review your reference list to ensure that it is complete and correct. If you have cited papers that have been retracted, please include the rationale for doing so in the manuscript text, or remove these references and replace them with relevant current references. Any changes to the reference list should be mentioned in the rebuttal letter that accompanies your revised manuscript. If you need to cite a retracted article, indicate the article’s retracted status in the References list and also include a citation and full reference for the retraction notice.

**We have reviewed our reference list to ensure that it is complete and correct.**

**Reviewers' comments:**

Reviewer's Responses to Questions

**Comments to the Author**

1. Is the manuscript technically sound, and do the data support the conclusions?

The manuscript must describe a technically sound piece of scientific research with data that supports the conclusions. Experiments must have been conducted rigorously, with appropriate controls, replication, and sample sizes. The conclusions must be drawn appropriately based on the data presented.

Reviewer #1: Yes

2. Has the statistical analysis been performed appropriately and rigorously?

Reviewer #1: Yes

3. Have the authors made all data underlying the findings in their manuscript fully available?

The [PLOS Data policy](http://track.editorialmanager.com/CL0/http:%2F%2Fwww.plosone.org%2Fstatic%2Fpolicies.action%23sharing/1/010f01955bbe83a4-9467b9ae-0532-45fa-8530-f18ed1df5e49-000000/_bn1PVovgpI3k5yCjXCX2G1N4H2U0MzurBUR5SgthNc=200) requires authors to make all data underlying the findings described in their manuscript fully available without restriction, with rare exception (please refer to the Data Availability Statement in the manuscript PDF file). The data should be provided as part of the manuscript or its supporting information, or deposited to a public repository. For example, in addition to summary statistics, the data points behind means, medians and variance measures should be available. If there are restrictions on publicly sharing data—e.g. participant privacy or use of data from a third party—those must be specified.

Reviewer #1: Yes

4. Is the manuscript presented in an intelligible fashion and written in standard English?

PLOS ONE does not copyedit accepted manuscripts, so the language in submitted articles must be clear, correct, and unambiguous. Any typographical or grammatical errors should be corrected at revision, so please note any specific errors here.

Reviewer #1: Yes

5. Review Comments to the Author

Please use the space provided to explain your answers to the questions above. You may also include additional comments for the author, including concerns about dual publication, research ethics, or publication ethics. (Please upload your review as an attachment if it exceeds 20,000 characters)

**Reviewer #1:** These researchers sought to determine if there is a difference in attendance at a six-week postpartum check among patients with and without epilespsy. Further if patients attended a SWC, were there differences in mental health outcomes or epilepsy-related complications. They used 4 administrative databases/registries to evaluate this question. Specific codelists were previously used to identify this population and healthcare visits. The statistical analysis seems appropriate using Poisson regression modeling as the outcome of a SWC if very common. Several models were fit to evaluate for possible important associations. Importantly, appropriate confounders were selected based on either prior evidence or hypotheses for confounding effects. Patients identified as having epilepsy were relatively similar to those without, apart from number of prior health care visits prior to pregnancy, and index of multiple deprivation.

Overall, the researchers used rigorous methodology to answer challenging but meaningful questions.
SPECIFIC POINTS:

INTROUDCTION:
Line 128 - Describe term A&E

**We have now described this term.**

METHODS:
Line 150 - Describe "Stillbirth" and how this is captured differently than miscarriage/clinical pregnancy loss

**We have amended the text in S1 Table to clarify this: “Since 1992, a stillbirth has been defined in the UK as a baby born dead after 24 weeks of pregnancy while a miscarriage includes all pregnancy losses from the time of conception until 24 weeks of pregnancy. In this study, a live birth or stillbirth was identified on the basis of having a CPRD pregnancy register outcome of 1, 2, 3, 11 or 12 OR having any ICD-10 or OPCS delivery related codes in HES APC or valid entry in a delivery related field in HES maternity (see the following files in** [**GitHub**](https://github.com/Fitzpatrickk/CPRD-Aurum-HES-codes_maternal-six-week-check_epilepsy_pregnancy/tree/main)**: ‘ICD-10_delivery codes’, ‘OPCS_delivery codes’ and `HES_Maternity_values_delivery’). See S1 Fig for details about how overlapping and implausibly close pregnancy records were dealt with. More details about the CPRD pregnancy register can be found in** [**https://www.cprd.com/sites/default/files/2022-05/CPRD%20Aurum%20PregnancyRegister_Documentation_v1.pdf**](https://www.cprd.com/sites/default/files/2022-05/CPRD%20Aurum%20PregnancyRegister_Documentation_v1.pdf)**”**

RESULTS:
Line 265-266 - the median age between the two groups is nearly identical. Similarly, prior prescription of prophylactic or emergency contraception, urinary/faecal incontinence and dyspareunia/pelvic pain are very similar between the groups. I assume these differences stated are based on a statistically significant p-value (which is not reported), and largely because of the large sample size rather than clinically meaningful differences between the groups.

**We agree that these differences are small and are unlikely to be clinically meaningful and have therefore now removed mention of these differences in the text.**

DISCUSSION:
Line 511 - I think you need to consider more strongly that GPs may not be assessing symptoms of dyspareunia and other symptoms of pelvic floor dysfunction adequately. This may be a reflection of a patient's discomfort with their GP, which is why they are not attending the SWC and therefore have a lower reported incidence. Alternatively, it is a poorly coded variable. Have there been any validation studies of the codes you are using to identify these symptoms with accuracy? I suspect these results are largely underrepresented in the way these data are collected.

**We have updated the paragraph in question to read: “Of importance to highlight, regardless of whether or not women had a SWC, the rates of general postpartum adverse health outcomes observed in the primary care records, at least for urinary and/or faecal urinary and/or faecal incontinence and dyspareunia and perineal and/or pelvic pain, appear to be on the low side given the prevalence of postpartum health problems that have been reported in the literature(25, 27). This could reflect the fact that previous studies have largely relied on surveys where postpartum women are asked directly about and self-report conditions or symptoms. This could pick up issues that women do not report to their GP for various reasons such as they are reluctant to, do not feel the need to, do not have the time to do, or have sought help for elsewhere. The guidance on administrating the SWC does list physical and pelvic issues among the topics to discuss, however we cannot tell if GPs are proactively asking women or if they assume women will report any significant problems. Women who do not have a SWC are perhaps even less likely to report problems like incontinence or dyspareunia, since it requires them to make an appointment to do so. We also cannot rule out we may have underestimated outcomes due to coding issues in the GP records. While comprehensive codelists were used to identify the outcomes, informed by clinical input and searching published articles and online codelist repositories, it’s possible the codes used had low sensitivity for identifying the outcomes although validation studies are needed to verify this.”**
